# ﻿Three new species of *Oocyclus* Sharp, 1882, with additional records from China (Coleoptera, Hydrophilidae, Laccobiini)

**DOI:** 10.3897/zookeys.1205.123579

**Published:** 2024-06-19

**Authors:** Feng-long Jia, Zu-qi Mai

**Affiliations:** 1 Institute of Entomology, Life Sciences School, Sun Yat-sen University, Guangzhou, 510275, Guangdong, China Sun Yat-sen University Guangzhou China; 2 Faculty of Environmental Sciences, Department of Ecology, Czech University of Life Sciences Prague, Czech Republic Czech University of Life Sciences Prague Prague Czech Republic

**Keywords:** Identification key, Oriental Region, water scavenger beetles, Xizang, Yunnan

## Abstract

Three new species of the water scavenger beetle genus *Oocyclus* Sharp, 1882 from China (*Oocyclusextensus***sp. nov.**, from Xizang, *O.latiorificialis***sp. nov.** and *O.ximaensis***sp. nov.** from Yunnan) are described and illustrated in detail. Additional faunistic data, illustrations of habitus and male genitalia, and a key to Chinese species are provided.

## ﻿Introduction

*Oocyclus* Sharp, 1882 is a pantropical genus of water scavenger beetle that is highly adapted to waterfalls and seepages. All known species are restricted to rock-face seeps, margins of waterfalls, and various kinds of vertical surfaces with water (Hebauer and Wang 1998; Short and Garcia 2010, Short et al. 2013; Short and Perkins 2004; Short and Swanson 2005; Toussaint and Short 2018). Currently, a total of 81 species have been described, of which 20 are known from the Oriental Region and 61 from the Neotropical Region (Minoshima 2009; Short 2009; Toussaint and Short 2018; Jordão et al. 2018; Santana et al. 2023; Alencar et al. 2022). Only six species have been recorded in China so far, of which *O.bhutanicus* Satô, 1976, cited from Taiwan by Hebauer and Wang (1998), needs to be confirmed (Short and Swanson 2005). Previous reports of *Oocyclus* in China have been limited to the southeastern part of the country (Hebauer and Wang 1998; Short and Jia 2011; Jia and Mate 2012). The distribution of the genus in other parts of China is still unknown.

Recently, we had the opportunity to visit some nature reserves in Yunnan and Xizang and discovered several *Oocyclus* species, of which three are described here as new to science. We also studied the *Oocyclus* collections at the Biological Museum, Sun Yat-sen University, and added information about the distribution of Chinese species.

## ﻿Materials and methods

Some of the specimens were dissected. Dissected male genitalia were transferred to a drop of distilled water, remaining membranes were removed under a compound microscope, and the cleaned genitalia were then mounted in a drop of soluble resin on a paper card attached below the respective specimen. For photography, the cleaned and relaxed male genitalia were placed in a drop of glycerin. Photographs of genitalia were taken using a Zeiss AxioCam HRc camera mounted on a Zeiss AX10 microscope with the Axio Vision SE64 software. These images were then stacked in Helicon Focus v. 7.0.2. Habitus photographs were taken using a Nikon DS-Ri2 mounted on a Nikon SMZ25; layers were captured and stacked in the NIS-Elements software. Habitat images were taken using Canon 7D digital camera. The generic characters are described in detail by Hansen (1991) and Short and Perkins (2004). Morphological terminology largely follows Hansen (1991) and Short and Perkins (2004). For a diagnosis of *Oocyclus* the reader is referred to Clarkson and Short (2012).

Examined specimens are deposited in the following collections:

**IZCAS** Chinese Academy of Sciences, Institute of Zoology, Beijing, China

**SYSU** Sun Yat-sen University, Guangzhou, China

## ﻿Results

### ﻿Descriptions of new species

#### ﻿ Oocyclus extensus sp. nov.

Taxon classificationAnimaliaColeopteraHydrophilidae

79F65058-208E-5495-8702-08125DD176DC

https://zoobank.org/2A7B3F2F-10FB-4431-8C33-67ED91F49C00

**Figure 1.**
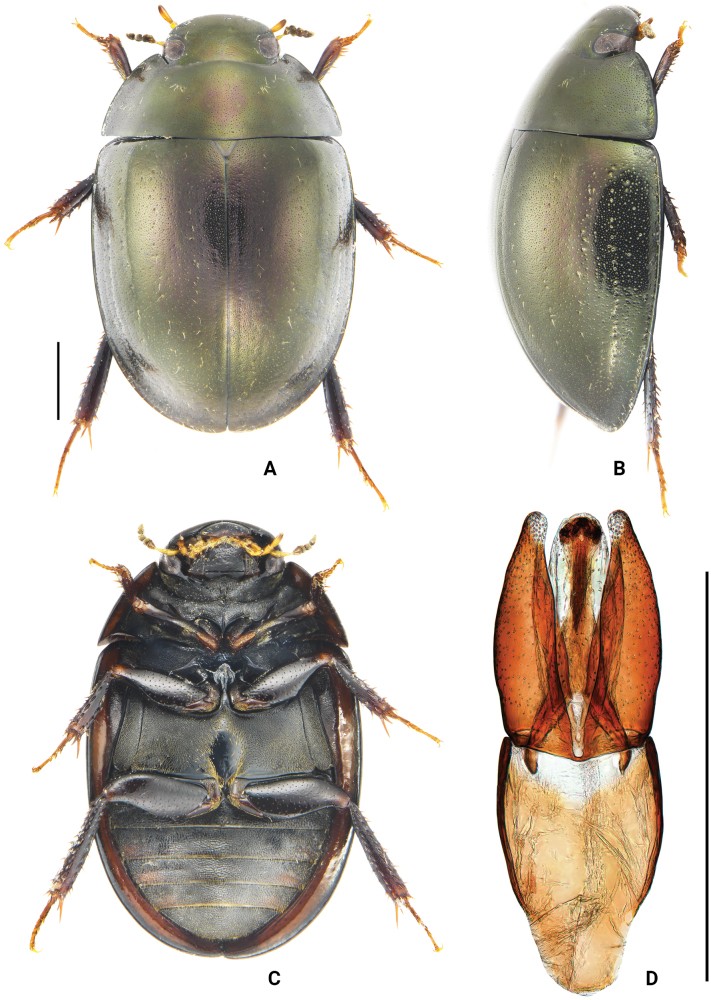
*Oocyclusextensus* sp. nov. **A** dorsal view **B** lateral view **C** ventral view **D** dorsal view of aedeagus. Scale bars: 1 mm.

Figs 1A–D , 4A–D , 6A , 7A , 8A

##### Type material.

***Holotype***: China • ♂; Xizang Autonomous Region, Xigazê, Dinggyê County, Zhêntang Town, on wet rock with a fine film of flowing water (西藏日喀则定结县陈塘镇流水岩壁表面); 27.8733°N, 87.4117°E; 2482 m elev.; 2023.VII.10; Zu-qi Mai, Cheng Liang & Yue-zheng Tu leg.; SYSU SYSBMZ2370001. ***Paratypes***: 8 ♂♂, 8 ♀♀(SYSU SYSBMZ2370002 to 0017), 2 ♂♂, 2 ♀♀ (IZCAS COLMZD2370001 to 0004); same data as the holotype.

##### Diagnosis.

Body large, length 5.3–6.2 mm, oblong-oval, and moderately convex. Dorsum black, with greenish luster under lateral illumination, slightly iridescent. Head, pronotum, and elytra with dense ground punctures consisting of extremely fine and moderately coarse punctures. Systematic punctures on labrum sparse and fine, not forming a continuous transverse row or groove. Posterolateral corners of pronotum angulate. Elytral suture slightly raised posteriorly; with 5 distinct rows of regular systematic punctures; lateral margins of elytra distinctly expanded outwards. Pseudepipleura wide from base to apex. Procoxae with sparse, spine-like setae scattered in fine pubescence. Meso- and metafemora without microsculpture on intervals of punctures. Abdominal ventrites with uniform pubescence over entire surface. Aedeagus (Fig. 1D) with parameres almost as long as median lobe; gradually narrowed from apical fourth to apex; apex of paramere slightly curved inwards and rounded. Median lobe slightly narrowed apically, gonopore situated apically, anterior margin of gonopore rounded.

##### Description.

***Form and color*** (Fig. 1A–C). Length 5.3–6.2 mm, width 3.4–4.0 mm; oblong-oval and moderately convex; elytra longer than wide. Dorsum black, with greenish luster under lateral illumination, slightly iridescent, more vividly colored when alive. Maxillary and labial palps yellowish brown, with last maxillary palpomere apically darkened. Antennae yellowish brown, with cupule (sixth antennomere) dark reddish brown or dark brown; club black. Ventral surface black. Epipleura, lateral margins of prosternum, and tarsomeres reddish brown; femora, tibiae, and sternites black or blackish brown. ***Head.*** Labrum, clypeus, and frons with both extremely fine and moderately coarse ground punctures; distance between punctures 0.2–3.0× width of 1 puncture. Systematic punctures on labrum sparse, not forming a continuous transverse row or groove, each systematic puncture with a long seta. Systematic punctures on clypeus almost undetectable. Frons with an irregular row of systematic punctures mesad of each eye, slightly larger than largest ground punctures and usually bearing long setae. Clypeus with a few very indistinct systematic punctures along anterolateral margins, slightly larger than surrounding ground punctures, and bearing short setae. Antennae with scape as long as antennomeres 2–5 combined, first 2 antennomeres of club subequal in length, and apical antennomeres slightly shorter than preceding 2 antennomeres combined. Maxillary palps short, subequal in length to the width of labrum, palpomere 2 slightly dilated, apical palpomeres ca 1.3× as long as penultimate. Labial palps ca 3/4× as the width of mentum. Mentum quadrate, anterior margin slightly convex; with coarse punctures on anterior and lateral portion, only with few punctures posteromedially. ***Thorax.*** Ground punctation on pronotum and elytra composed of extremely fine and moderately coarse punctures evenly mixed and distributed. Pronotal systematic punctures present, ca 1.5× the size of ground punctation and set with a fine seta, sometimes partially blending with coarser ground punctures; anterior and posterior series each forming an irregular row. Lateral margins of pronotum set with a few sparsely distributed setiferous punctures. Pronotum with anterior and lateral marginal rims, posterior marginal rim absent. Posterolateral corners of pronotum angulate. Prosternum moderately tectiform, with median carina along entire length, with a small blunt tooth anteriorly. Elytra with 5 distinct rows of punctures, diameter of systematic punctures ca 2× as wide as the coarse ground punctures; lateral margin of elytra distinctly expanded outwards, especially in posterior half; elytral suture slightly raised but easily detectable. Pseudepipleura wide throughout. Mesoventral process with lateral extensions sloping evenly downward. Metaventrite with an oval glabrous area posteromedially, slightly longer than wide, length of glabrous area as long as the total length of metaventrite. Pro- and mesocoxae densely pubescent; procoxae with sparse spine-like setae. Ventral surface of profemora densely pubescent at basal fifth, remainder scattered with fine punctures, interstices without microsculpture; meso- and metafemora glabrous, with coarse punctures and without microsculpture. Protibiae with several spines on dorsal face. Fifth tarsomere of pro- and mesotarsus subequal in length to the preceding 4 tarsomeres combined. Fifth metatarsomere equal in length to second tarsomere. ***Abdomen.*** Abdominal ventrites 1–5 with uniform pubescence, longest setae about as long as the setae around the metaventral glabrous area. Fifth ventrite entire. ***Aedeagus*** (Fig. 1D). Phallobase with basal 2/3 arcuate, manubrium gradually narrowed at posterior 1/3 and rounded posteriorly. Parameres almost as long as phallobase, widest at the base, arcuate medially outwards, gradually narrowed from apical fourth to apex; apex of paramere slightly curved inwards and rounded. Median lobe slightly narrowed apically, gonopore triangular, apically situated, and with rounded anterior margin.

##### Remarks.

This species is similar to *Oocyclusrupicola* Minoshima, 2009 from Laos. It can be distinguished from *O.rupicola* by its on average larger body size (length 5.3–6.2 mm, width 3.4–4.0 mm vs length 4.93–5.85 mm, width 3.03–3.53 in *O.rupicola*), sparse, fine systematic punctures on labrum which do not form a continuous transverse row (vs forming a row of coarse punctures in *O.rupicola*), and rounded apex of the paramere of the aedeagus (vs narrowed and obliquely truncate inwards in *O.rupicola*) (Minoshima 2009).

##### Etymology.

This species is named *extensus*, Latin, meaning “stretched out” and referring to the outwardly expanded elytra.

##### Biology.

(Fig. 4A–D) The examined specimens were collected on exposed, seeping rock surfaces on the valley edge. They were together with other species of the family Hydrophilidae: *Oocyclusbhutanicus* Satô, 1979, *Agraphydrusnepalensis* Komarek, 2018, *Laccobiusregalis* Knisch, 1924, and *Coelostomagentilii* Jia, Aston & Fikáček, 2014. Adults hide in rock crevices during the day and are active at night.

##### Distribution.

(Fig. 9) China (Xizang). Only known from type locality. Zhêntang Town is in a valley on the southern side of the central Himalayas and is a border town on the China–Nepal border and lies on the Pum Qu River.

#### ﻿ Oocyclus latiorificialis sp. nov.

Taxon classificationAnimaliaColeopteraHydrophilidae

C5F4E4DD-BB37-5BA6-B5C5-94953C815B47

https://zoobank.org/7793C4B1-3AAC-4664-9D8F-B8E4EE74BBDC

**Figure 2.**
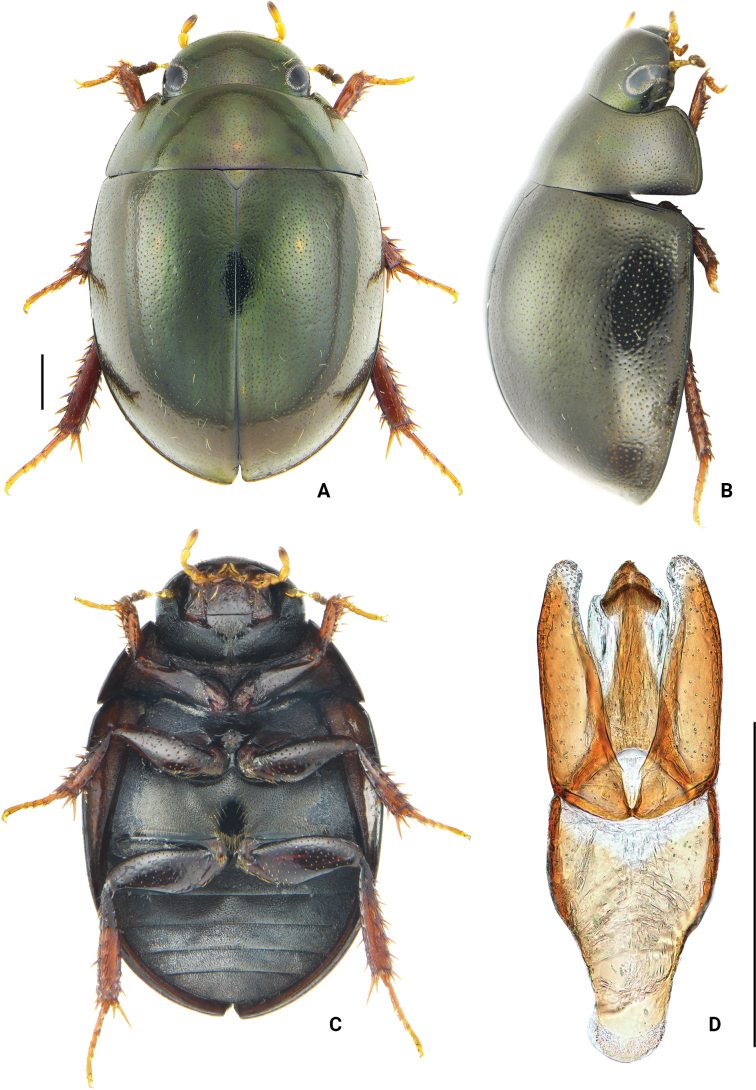
*Oocycluslatiorificialis* sp. nov. **A** dorsal view **B** lateral view **C** ventral view **D** dorsal view of aedeagus. Scale bars: 0.5 mm.

Figs 2A–D , 6E , 7E , 8E

##### Type material.

***Holotype***: China • ♂; Yunnan Province, Nujiang Lisu Autonomous Prefecture, Lushui County, Pianma Village, on wet rock with fine flowing water (怒江傈僳族自治州泸水市片马村流水岩壁); 26.014039°N, 98.650640°E; 2131 m elev.; 2021.V.17; Zhuo-yin Jiang, Zhen-ming Yang & Zu-qi Mai leg.; SYSU SYSBMZ2370018. ***Paratypes***: 1 ♂; same data as the holotype; SYSU SYSBMZ2370019. • 16 unsexed spec.; Yunnan Prov., Lushui County, Yaojiaping (泸水县姚家坪); 26.1°N, 98.71°E; 2424 m elev.; 2016.V.17; Yu-dan Tang & Rui-juan Zhang leg.; SYSU SYSBMZ2370020 to 0035.

##### Diagnosis.

Body medium-sized, length 4.1–4.3 mm, oval, and rather convex. Dorsum black, with distinct greenish luster under lateral illumination, slightly iridescent. Head, pronotum, and elytra with dense ground punctures consisting of extremely fine and moderately coarse punctures. Systematic punctures on labrum moderately dense, forming a nearly continuous transverse row. Posterolateral corners of pronotum evenly rounded. Elytral suture not raised; without distinct rows of systematic punctures; lateral margins of elytra not expanded outwards. Pseudepipleura narrowed posteriorly. Procoxae without distinct, spine-like setae in fine pubescence. Meso- and metafemora with fine microsculpture on intervals of punctures. Abdominal ventrites with uniform pubescence over entire surface. Aedeagus (Fig. 2D) with parameres almost as long as median lobe; inner margin of paramere sinuate in dorsal view and distinctly, subapically curved inward; apex of paramere broadly rounded. Median lobe parallel-sided medially, slightly widened at site of gonopore; gonopore situated apically; anterior margin of gonopore pointed.

##### Description.

***Form and color*** (Fig. 2A–C). Length 4.1–4.4 mm width 2.4–2.7mm, oval, rather convex; elytra longer than wide. Dorsum black, with distinct greenish luster under lateral illumination, slightly iridescent, more vividly colored when alive. Maxillary palps yellow, with apex black; labial palpi yellow. Mentum and stipes light to reddish brown, paler than ventral face of head. Legs, epipleura, lateral margins of prosternum, and posterior margin of each ventrite light brown to dark reddish brown, with remainder of venter darker reddish brown. ***Head.*** Labrum, clypeus, and frons with both extremely fine and moderately coarse ground punctures, distance between punctures 0.5–4.0× as the width of 1 puncture. Systematic punctures on labrum moderately dense, forming a nearly continuous transverse row, with moderately long setae. Frons with an irregular row of systematic punctures mesad of each eye, bearing fine erect setae. Clypeus with a few very indistinct systematic punctures along anterolateral margins, slightly larger than surrounding ground punctures and bearing short setae. Antennae with scape subequal in length to antennomeres 2–5 combined; first two antennomeres of club subequal in length, and apical antennomere slightly longer than two preceding antennomeres combined. Maxillary palps short, subequal in length to the width of labrum; palpomere 2 slightly dilated, apical palpomere ca 1.3 as long as penultimate. Labial palpi ca 3/4 width of mentum. Mentum quadrate, anterior margin slightly convex, bearing very fine and scattered punctures. ***Thorax.*** Ground punctation on pronotum and elytra composed of extremely fine and moderately coarse punctures evenly mixed and distributed. Pronotal systematic punctures present, ca 1.5–2.0× size of ground punctation and set with a fine seta, sometimes partially blending with coarser ground punctures; anterior and posterior series each forming an irregular row. Lateral margins of pronotum set with a few sparsely distributed setiferous punctures. Pronotum with anterior and lateral marginal rims, posterior marginal rim absent. Posterolateral corners of pronotum evenly rounded. Prosternum moderately tectiform, with median carina along entire length but without distinct anteromedial tooth. Elytra without distinct rows of larger punctures, systematic punctures almost as coarse as coarser ground punctures, distinguished by the presence of fine, short setae; lateral margins of elytra not expanded outwards; elytra suture not raised. Pseudepipleura narrowed posteriorly. Mesoventral process with lateral extensions sloping evenly downward. Metaventrite posteromedially with an oval glabrous area, slightly longer than wide, length of glabrous area about 2/3 of the total length of metaventrite. Pro- and mesocoxae densely pubescent, without distinct spine-like setae. Ventral surface of profemora densely pubescent at basal 1/5, remainder scattered with fine punctures, interstices with fine microsculpture; meso- and metafemora glabrous, with coarse punctures and fine microsculpture. Protibiae with several spines on dorsal face. Fifth tarsomere of pro- and mesotarsus subequal in length to the preceding 4 tarsomeres combined. Fifth metatarsomere equal in length to second tarsomere. ***Abdomen.*** Abdominal ventrites 1–5 with uniform pubescence, longest setae about as long as the setae around the metaventral glabrous area. Fifth ventrite entire. ***Aedeagus*** (Fig. 2D). Phallobase parallel-sided to slightly arcuate at basal half, manubrium gradually narrowed at posterior 1/2 of phallobase and rounded posteriorly. Parameres slightly longer than phallobase, widest at the base and gradually narrowed, inner margin of paramere sinuate in dorsal view and distinctly curved subapically; apices broadly rounded. Median lobe almost as long as parameres, parallel-sided medially, and slightly widened at site of gonopore; gonopore triangular, situated apically, and with pointed anterior margin.

##### Remarks.

This species is very similar to *O.bhutanicus* Satô, 1979, but can be easily distinguished by meso- and metafemora with fine microsculpture on intervals of punctures (vs lacking microsculpture in *O.bhutanicus*), aedeagus with median lobe as long as parameres and almost parallel-sided medially, slightly widened at level of gonopore (vs median lobe shorter than parameres and gradually narrowed from base to apex in *O.bhutanicus*), anterior margin of gonopore pointed (vs rounded in *O.bhutanicus*), and inner margin of paramere sinuate in dorsal view and distinctly curved subapically (vs inner margin of paramere almost straight in dorsal view in *O.bhutanicus*). This species also shares diagnostic features with *O.ximaensis* sp. nov., but it can be distinguished from the latter by dorsum with distinct greenish luster under lateral illumination (vs greenish luster weaker in *O.ximaensis*), meso- and metafemora with fine microsculpture (lacking microsculpture in *O.ximaensis*), and aedeagus with median lobe almost parallel-sided medially, slightly widened at level of gonopore (vs median lobe gradually narrowed from base to apex in *O.ximaensis*).

##### Etymology.

Species name is combination of Latin *latus*, “wide”, and *orificialis*, “orifice”, referring to the widely open gonopore.

##### Biology.

This species was founding living on wet rock surface at the margins of a waterfall.

##### Distribution.

(Fig. 9) China (Yunnan). Only known from type locality.

#### ﻿ Oocyclus ximaensis sp. nov.

Taxon classificationAnimaliaColeopteraHydrophilidae

0FB286BF-8C12-536E-8074-0C3D3C1714DD

https://zoobank.org/2A925CD2-EC80-4A22-971E-7C0024471B48

**Figure 3.**
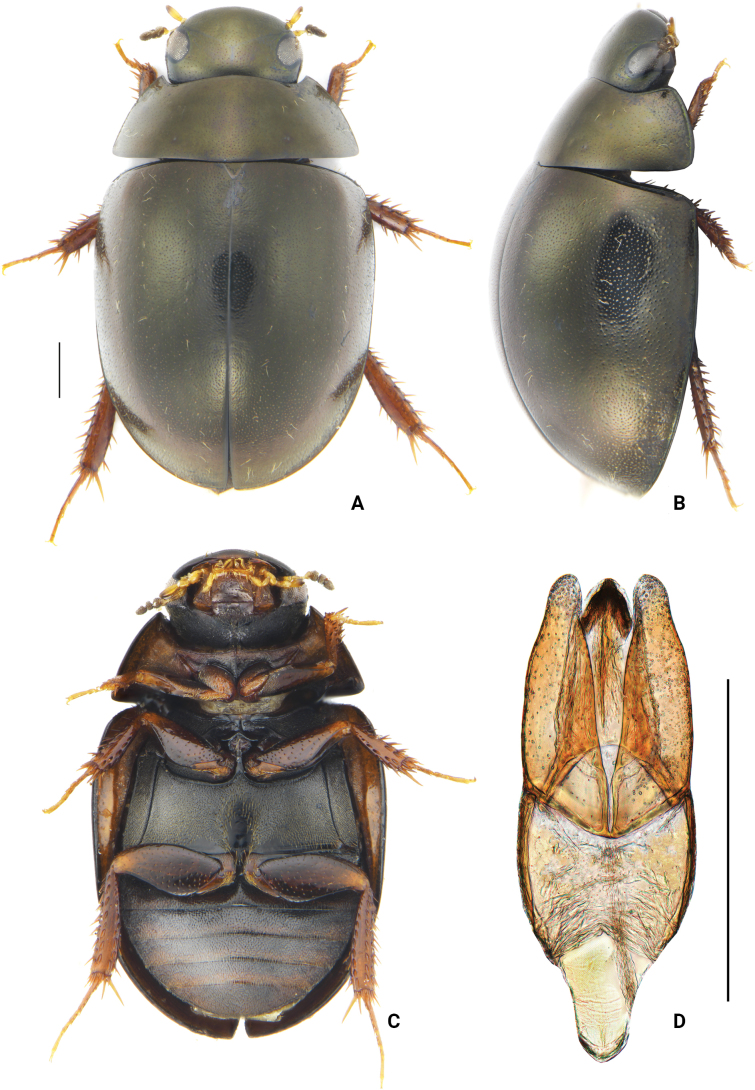
*Oocyclusximaensis* sp. nov. **A** dorsal view **B** lateral view **C** ventral view **D** dorsal view of aedeagus. Scale bars: 0.5 mm.

Figs 3A–D , 5A–C , 6F , 7F , 8F

##### Type material.

***Holotype***: China • ♂; Yunnan Prov., Dehong Dai and Jingpo Autonomous Prefecture, Yingjiang County, Xima Town, Xingyun Secondary power stations (星云二级电站), at seepage rock wall by the river, 24.7854°N, 97.6472°E, 1021 m, 2022.VIII.19, Zu-qi Mai, Yu-chen Zheng & Yue-zheng Yu leg.; SYSU SYSBMZ2370036. ***Paratypes***: 2 ♂♂, 5 ♀♀ (SYSU SYSBMZ2370037 to 0043), same data as the holotype.

##### Diagnosis.

Body medium-sized, length 3.8–4.4 mm, oval, and rather convex. Dorsum black, with weak greenish luster, slightly iridescent. Head, pronotum, and elytra with dense ground punctures consisting of extremely fine, moderately coarse punctures. Systematic punctures on labrum moderately dense, forming a nearly continuous transverse row. Posterolateral corners of pronotum evenly rounded. Elytra suture not raised, without distinct rows of systematic punctures; lateral margins of elytra not expanded outwards. Pseudepipleura narrowed posteriorly. Procoxae without distinct, spine-like setae in fine pubescence. Meso- and metafemora without microsculpture on intervals of punctures. Abdominal ventrites with uniform pubescence over entire surface. Aedeagus (Fig. 3D) with parameres almost as long as median lobe; inner margin of paramere slightly sinuate in dorsal view and hardly curved subapically; apex of paramere rounded. Median lobe gradually narrowed from base to apex; gonopore situated apically; anterior margin of gonopore pointed.

##### Description.

***Form and color*** (Fig. 3A–C). Length 3.8–4.4 mm, width 2.3–2.7 mm, oval, rather convex; elytra longer than wide. Dorsum black with weak greenish luster under lateral illumination, slightly iridescent, more vividly colored when alive. Maxillary palps yellow with apex black, labial palpi yellow. Mentum and stipes yellowish brown, paler than ventral face of head. Legs, epipleura, lateral margins of prosternum, and posterior margin of each ventrite light brown to yellowish brown, with remainder of venter darker yellowish brown. ***Head.*** Labrum, clypeus, and frons with both extremely fine and moderately coarse ground punctures, distance between punctures 1.0–3.5× width of 1 puncture. Systematic punctures on labrum moderately dense, forming a nearly continuous transverse row setting with moderately long setae. Frons with an irregular row of systematic punctures mesad of each eye, bearing fine, erect setae. Clypeus with a few very indistinct systematic punctures along anterolateral margins, slightly larger than surrounding ground punctures, and bearing short setae. Antennae with scape subequal in length to antennomeres 2–5 combined, first 2 antennomeres of club subequal in length, and apical antennomere slightly longer than 2 preceding antennomeres combined. Maxillary palps short, subequal in length to the width of labrum, palpomere 2 slightly dilated, apical palpomeres ca 1.3 as long as penultimate. Labial palps ca 3/4 width of mentum. Mentum quadrate, anterior margin slightly convex, bearing very fine and scattered punctures. ***Thorax.*** Ground punctation on pronotum and elytra composed of extremely fine and moderately coarse punctures evenly mixed and distributed. Pronotal systematic punctures present, ca 1.5–2.0× size of ground punctation and set with a fine seta, sometimes partially blending with coarser ground punctures; anterior and posterior series each forming an irregular row. Lateral margins of pronotum set with a few sparsely distributed setiferous punctures. Pronotum with anterior and lateral marginal rims, posterior marginal rim absent. Posterolateral corners of pronotum evenly rounded. Prosternum moderately tectiform, with median carina along entire length but without distinct anteromedial tooth. Elytra without distinct rows of larger punctures, systematic punctures almost as coarse as coarser ground punctures, distinguished by the presence of fine and short setae; lateral margins of elytra not expanded outwards; elytra suture not raised. Pseudepipleura narrowed posteriorly. Mesoventral process with lateral extensions sloping evenly downward. Metaventrite posteromedially with an oval, glabrous area, slightly longer than wide, length of glabrous area ca 2/3 of total length of metaventrite. Pro- and mesocoxae densely pubescent; without distinct spine-like setae. Ventral surface of profemora densely pubescent at basal 1/5, remainder scattered with fine punctures, interstices smooth, without microsculpture; meso- and metafemora glabrous, with coarse punctures and without microsculpture. Protibiae with several spines on dorsal face. Fifth tarsomere of pro- and mesotarsus subequal in length to the preceding 4 tarsomeres combined. Fifth metatarsomere equal in length to second tarsomere. ***Abdomen.*** Abdominal ventrites 1–5 with uniform pubescence; longest setae about as long as setae around metaventral glabrous area. Fifth ventrite entire. ***Aedeagus*** (Fig. 3D). Phallobase slightly arcuate at basal 2/3; manubrium gradually narrowed at posterior 1/3 and rounded posteriorly. Parameres slightly longer than phallobase, widest at the base and gradually narrowed; inner margin of paramere slightly sinuate in dorsal view and hardly subapically curved; apex rounded. Median lobe almost as long as parameres, gradually narrowed from base to apex; gonopore triangular, apically situated; anterior margin of gonopore pointed.

##### Remarks.

This species is very similar to *O.bhutanicus* Satô, 1979, but it can be distinguished from the latter by dorsum with weak greenish luster under lateral illumination (vs distinct greenish luster in *O.bhutanicus*), aedeagus with median lobe as long as parameres (vs shorter than parameres in *O.bhutanicus*), anterior margin of gonopore pointed (rounded in *O.bhutanicus*).

##### Etymology.

This species is named after Xima Town, where the type locality is located.

##### Biology.

(Fig. 5A–C) This species was found on the surface of vertical stone walls with seepage on the side of the river valley.

**Figure 4.**
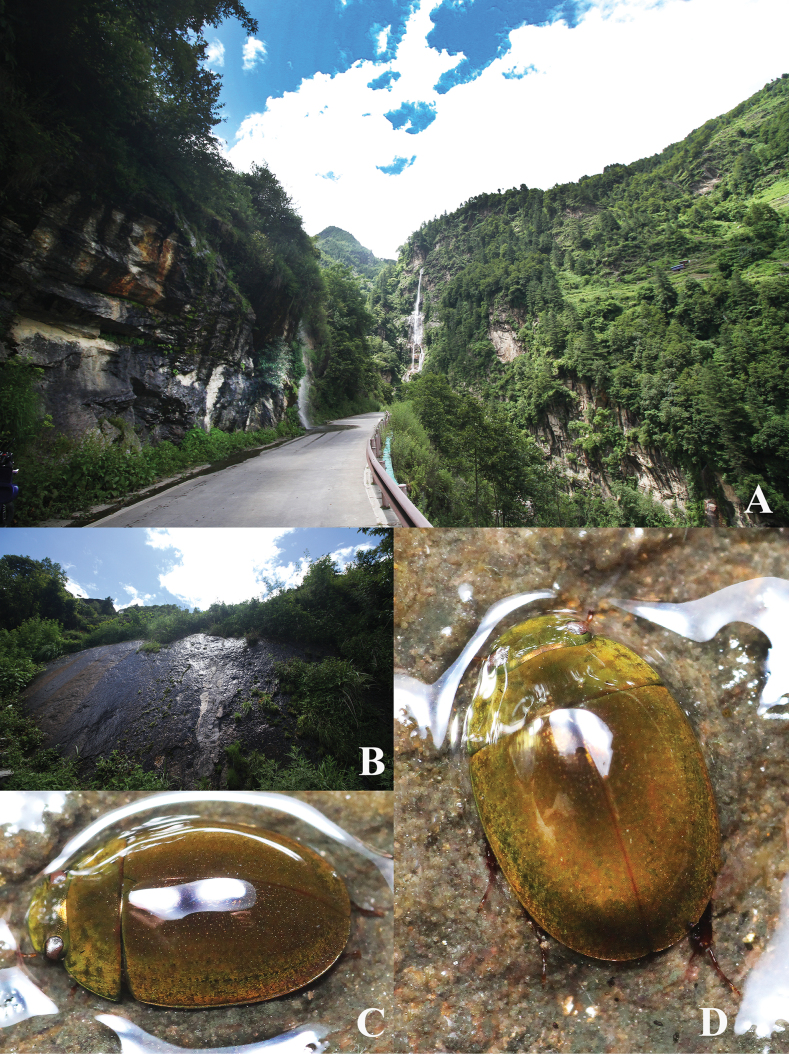
Habitats of *Oocyclusextensus* sp. nov. **A** valley at Zhêntang Town (Xizang), roadside with waterfalls **B** wet rock with fine flowing water **C, D** adults at night.

**Figure 5.**
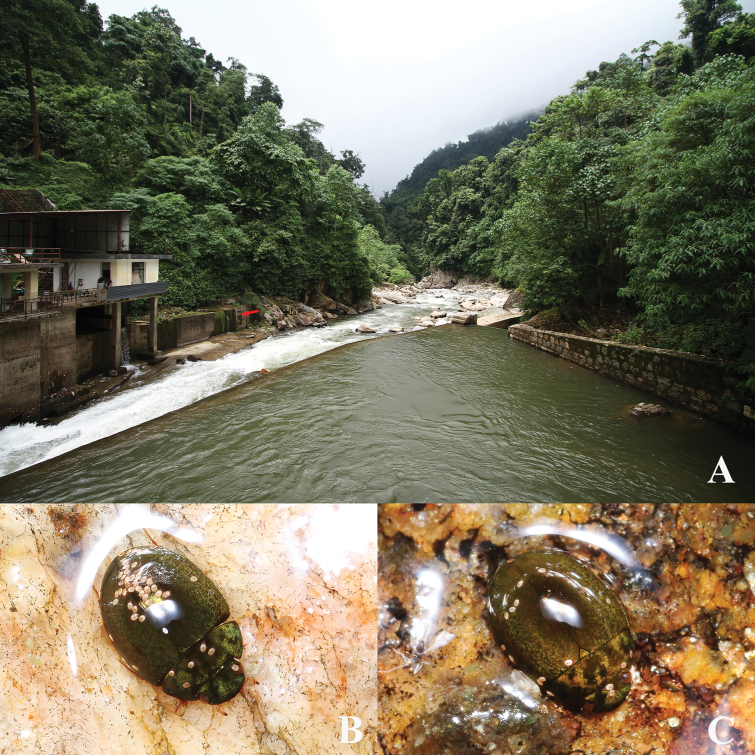
Habitat of *Oocyclusximaensis* sp. nov. **A** river valley with forests on both sides in Xima Town (Yunnan); the red arrow indicates the rock wall with flowing water **B, C** adults at night.

##### Distribution.

(Fig. 9) China (Yunnan). Only known from type locality.

### ﻿Additional faunistic records for China

#### ﻿ Oocyclus bhutanicus

Taxon classificationAnimaliaColeopteraHydrophilidae

Satô, 1979

8E858B7A-B4F1-59AC-AE4B-C136BDCE992F

Figs 6D , 7D , 8D

##### New material examined.

5 ♂♂, 3 ♀♀; China• Xizang Autonomous Region, Xigazê, Dinggyê County, Zhêntang Town, on wet rock with fine flowing water (西藏日喀则定结县陈塘镇流水岩壁表面), 2482 m, 27.8733°N, 87.4117°E, 2023.VII.10, Zu-qi Mai.; SYSU BJSB438001 • 1 ♂, 3 ♀♀ (SYSU), Xizang, Medog County, 80K, 1371 m, 27.8733N, 95.4673E, 2023.VI.15, Zu-qi Mai leg.; SYSU BJSB438002 to 005.

**Figure 6.**
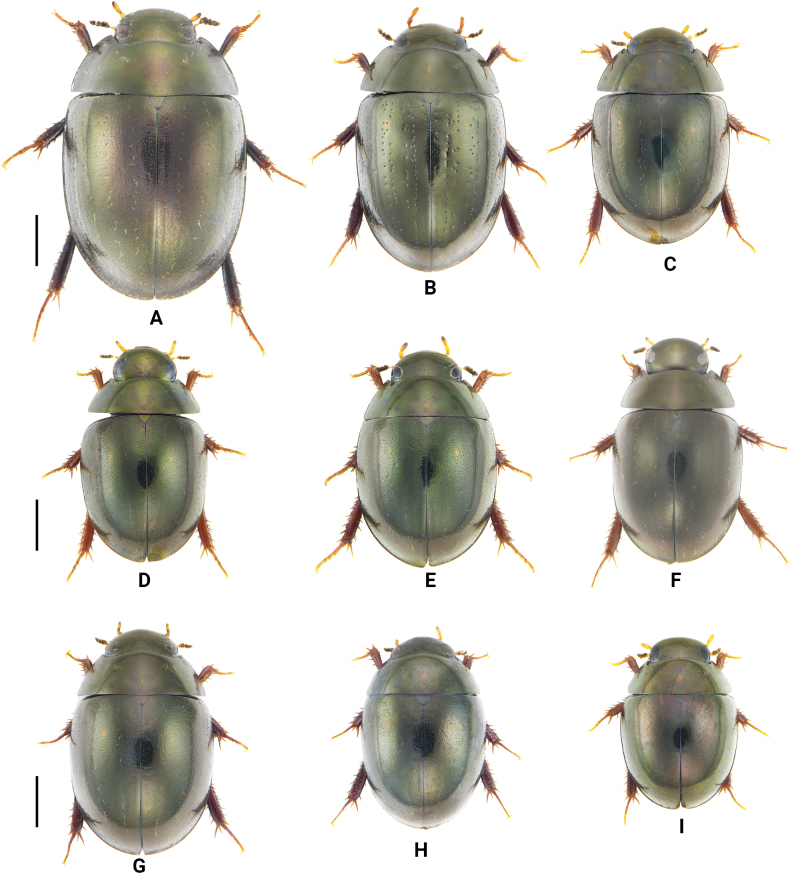
Dorsal view of Chinese *Oocyclus* spp. **A***O.extensus* sp. nov. **B***O.magnificus* Hebauer & Wang, 1998 **C***O.sumatrensis* Orchymont, 1932 **D***O.bhutanicus* Satô, 1979 **E***O.latiorificialis* sp. nov. **F***O.ximaensis* sp. nov. **G***O.fikaceki* Short & Jia, 2011 **H***O.shorti* Jia & Maté, 2012 **I***O.dinghu* Short & Jia, 2011. Scale bars: 1 mm.

**Figure 7.**
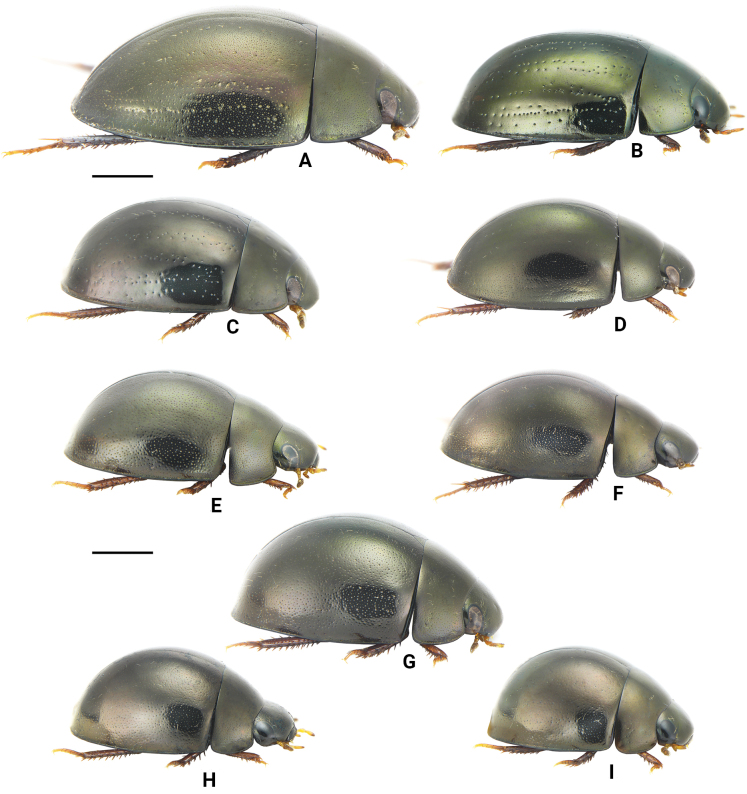
Lateral view of Chinese *Oocyclus* spp. **A***O.extensus* sp. nov. **B***O.magnificus* Hebauer & Wang, 1998 **C***O.sumatrensis* Orchymont, 1932 **D***O.bhutanicus* Satô, 1979 **E***O.latiorificialis* sp. nov. **F***O.ximaensis* sp. nov. **G***O.fikaceki* Short & Jia, 2011 **H***O.shorti* Jia & Maté, 2012 **I***O.dinghu* Short & Jia, 2011. Scale bars: 1 mm.

**Figure 8.**
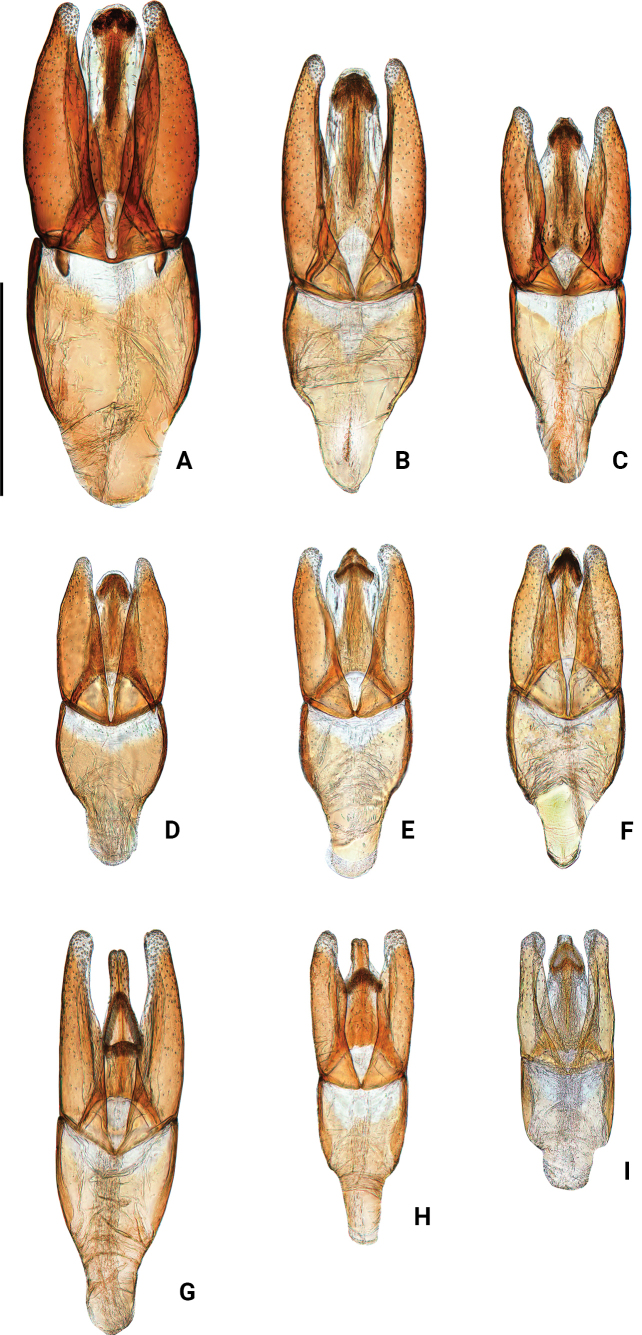
Aedeagus of Chinese *Oocyclus* spp. (dorsal view) **A***O.extensus* sp. nov. **B***O.magnificus* Hebauer & Wang, 1998 **C***O.sumatrensis* Orchymont, 1932 **D***O.bhutanicus* Satô, 1979 **E***O.latiorificialis* sp. nov. **F***O.ximaensis* sp. nov. **G***O.fikaceki* Short & Jia, 2011 **H***O.shorti* Jia & Maté, 2012 **I***O.dinghu* Short & Jia, 2011. Scale bar: 0.5 mm.

##### Distribution.

**Figure 9.**
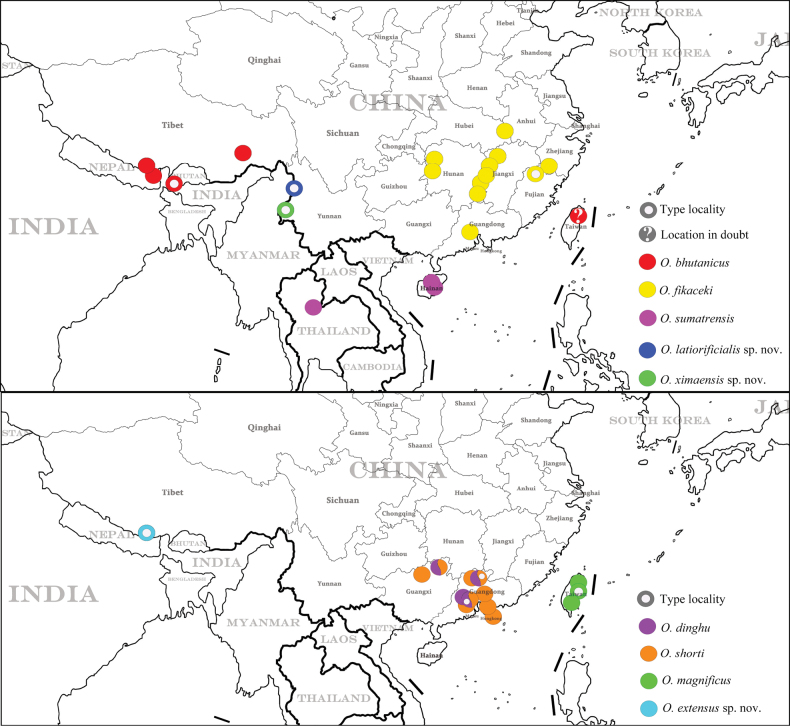
Distribution maps of Chinese *Oocyclus* spp.

(Fig. 9) China (Xizang), Bhutan, Nepal. **Newly recorded from Xizang.** Records from Thailand and China (Taiwan) by Hebauer and Wang (1998) need confirmation (Short and Swanson 2005).

#### ﻿ Oocyclus dinghu

Taxon classificationAnimaliaColeopteraHydrophilidae

Short & Jia, 2011

0FA58140-FB51-5EE6-B29B-7BA37EDCA200

Figs 6I , 7I , 8I

##### New material examined.

12 unsexed spec.; China• Guangdong Prov., Fengkai County, Heishiding Natural Reserve (广东省封开黑石顶自然保护区), 23.31°N, 111.52°E, 2014.IX.20–22, Ren-chao Lin, Feng-long Jia & Yu-dan Tang leg; SYSU BJSB439001. • 2 unsexed spec.; Guangdong, Dinghushan Natural Reserve (广东鼎湖山自然保护区), 1958, Cui-ying Li leg. (with label *Oocycluslatus*).; SYSU BJSB439002 to 003. • 1 ♂, 2 ♀♀; Guangxi Prov., Longsheng, Jiangdi (龙胜江底乡), 2013.IV.13–17, Hai-dong Chen leg.; SYSU BJSB4381006 to BJSB4381008.

##### Distribution.

(Fig. 9) China (Guangdong, Guangxi). **Newly recorded from Guangxi**.

#### ﻿ Oocyclus fikaceki

Taxon classificationAnimaliaColeopteraHydrophilidae

Short & Jia, 2011

23A0CD0E-5893-552E-8021-3D555223E397

Figs 6G , 7G , 8G

##### New material examined.

China • **Zhejiang**: 2 ♂♂, 3 ♀♀, 14 unsexed spec.; Longquan, Fengyangshan Natural Reserve (龙泉凤阳山自然保护区), on wet rock, N.R. ca 1150m, 27°54′51″N, 119°11′56″E, 2018.IV.28, Yin & Miao.; SYSU. • **Hubei**: 2 ♂♂, 34 unsexed spec.; Dabieshan Mountian, Taohuachong mount (大别山脉桃花冲), 30°50.9′N, 116°1.7′E, 2014.VI.25, Zhen-hua Liu leg.; SYSU. • **Hunan**: 3 ♂♂, 6 ♀♀, 427 unsexed spec.; Zhuzhou Prefecture, Yanling County, Taoyuandong Natural Reserve, Zhulian waterfall (株洲市炎陵县桃源洞珠帘瀑布), 2014.V.26,Xiao-hua Chen, Ren-chao Lin & Chang Pan leg.; SYSU. • 168 unsexed spec.; Guidong County, Bamianshan Natural Reserve (桂东县八面山自然保护区),25°58′21″N, 113°42′37″E, 973m, 2015.VI.15, Ren-chao Lin & Yu-dan Tang leg.; SYSU. • 30 unsexed spec.; Guzhang County, Gaowangjie National Natural Reserve (古丈县高望界国家自然保护区), 28°29.898′N, 110°3.575′E, 1053m, 2017.VI.21, Feng-long Jia leg.; SYSU. • 23 unsexed spec.; Huaihua Prefecture, Mayang County, Lancun (怀化麻阳县兰村乡), 27°46′18″N, 109°51′51″E, 2017.VIII.14, Wei-cai Xie & Shi-shuai Wang leg.; SYSU. • **Jiangxi**: 32 unsexed spec.; Jing’an County, Daqishan forest farm (静安县大杞山林场), 28.67°N, 115.07°E, 350m, 2014.VII.16, Ren-chao Lin leg.; SYSU. • 1 ♂; Jing’an County, Sanzhaolun, Baishuidong scenic area (静安县三爪仑乡白水洞景区), 29.04°N, 115.11°E, 660m, 2014.VII.22, Ren-chao Lin leg.; SYSU. • 5 unsexed spec., Anfu County, Wugongshan (安福县武功山), 27.33°N, 114.23°E, 400m, 2014.VII.24; SYSU. • 1 unsexed spec.; Jing’an County, Guanyinyan (静安县观音岩), 29.04°N, 115.14°E, 690m, 2014.VII.20, Ren-chao Lin; SYSU.

##### Distribution.

(Fig. 9) China (Fujian, Guangdong, Hubei, Hunan, Jiangxi, Zhejiang). **Newly recorded from Hubei, Hunan, and Zhenjiang**.

#### ﻿ Oocyclus magnificus

Taxon classificationAnimaliaColeopteraHydrophilidae

Hebauer & Wang, 1998

87A0F08B-E20F-51A7-B6EB-59AE62164EFA

Figs 6B 7B, 8B

##### New material examined.

3 ♂♂, 4 ♀♀; China • Taiwan, Taidong, Hairui, Lidao village (台东海瑞乡利稻), 1000m, 2017.V.13, Wen-yi Zhou leg.; SYSU. MZPC0050 to 0056. • 2 ♂♂, 1 ♀, 10 unsexed spec.; China • Taiwan, Lala Shan Mt. (between Fuxing town, Taoyuan County and Wulai town, Taibei County) (拉拉山,桃园县复兴镇和台北县乌来镇之间), 2006.IV.26, Living leg.; SYSU.

##### Distribution.

(Fig. 9) China (Taiwan).

#### ﻿ Oocyclus shorti

Taxon classificationAnimaliaColeopteraHydrophilidae

Jia & Maté, 2012

27AA6452-A03C-55CA-B6D1-5E5A467EA612

Figs 6H , 7H , 8H

##### New material examined.

China • **Guangdong**: 3 ♂♂ 2 ♀♀, 47 unsexed spec.; Huizhou Prefecture, Longmen County, Nankunshan, Zhongpingwei Village (惠州龙门县南昆山中坪尾村), 23.6224°N, 113.8660°E, 639.0m, 2021.IX.26, Wei-cai Xie, Zhuo-yin Jiang & Zu-qi Mai leg.; SYSU. • 47 unsexed spec.; Fengkai County, Heishiding Natural Reserve (封开县黑石顶保护区), 23°31′N, 113°52′E, 2014.XI.20–22, Ren-chao Lin, Feng-long Jia & Yu-dan Tang et al. leg.; SYSU. • 8 unsexed spec.; Renhua County, Danxiashan, Zhanglaofeng (仁化县丹霞山长老峰), 2012.V.30, Feng-long Jia leg.; SYSU. • 3 unsexed spec.; Danxiashan, Jinshiyan (丹霞山锦石岩), 2021.VI.08, Feng-long Jia leg.; SYSU. • 1 ♂.; Danxiashan, Xianglong Lake (丹霞山翔龙湖), 2012.VIII.08, Feng-long Jia leg.; SYSU. • 45 unsexed spec.; Danxiashan, near Danxian Resort (丹霞山丹霞山庄), on wet rock, 2016.VI.08, Feng-long Jia leg.; SYSU. • 20 unsexed spec.; Shenzhen, Maluanshan Mt., (深圳马峦山), 2014.VI.13, Feng-long Jia & Wei-cai Xie leg.; SYSU. • 2 ♂♂, 1 ♀, 28 unsexed spec.; Shaoguan Prefecture, Chebaling National Natural Reserve (韶关车八岭国家自然保护区), 23°14′46″N, 113°33′56″E, 496m, 2017.V.28–29, Feng-long Jia, Shi-shuai Wang & Zu-long Liang leg.; SYSU. • 1 ♂; Foshan Prefecture, Gaoming County, Yangmei town (高明市杨梅镇),2006.IV.23–26, Feng-long Jia leg.; SYSU. • 16 unsexed spec.; Qingyuan Prefecture, on wet rock of roadside from Lianzhou city to Tianlong Gorge, Datongshan Natural Reserve (连州至大东山自然保护区天龙峡路边潮湿石壁), 2013.IV.18, Feng-long Jia leg.; SYSU. • 3 ♂♂, 2 ♀♀, 40 unsexed spec.; Nanling, Dadongshan Natural Reserve (大东山自然保护区), 2013.IV.19–22, Feng-long Jia.; SYSU. • **Guangxi**: 2 ♂♂, 4 ♀♀, 64 unsexed spec.; Jiuwandashan, Yangmeiao village (九万大山杨梅坳), 25°11′42″N, 108°28′51″E, 1183m, 2015.VII.20, Ren-chao Lin & Yu-dan Tang; SYSU. • 2 ♂♂, 4 ♀♀, 28 unsexed spec.; Longsheng County, Jiangdi (龙胜江底乡), 2013.IV.13–17, Hai-dong Chen leg.; SYSU.

##### Distribution.

(Fig. 9) China (Guangdong, Guangxi, Hongkong). **Newly recorded from Guangxi.**

#### ﻿ Oocyclus sumatrensis

Taxon classificationAnimaliaColeopteraHydrophilidae

Orchymont, 1932

A7FD8B0D-45B3-580B-85E7-EC9D4380F641

Figs 6C , 7C , 8C

##### New material examined.

2 ♂♂, 1 ♀, 11 unsexed spec.; China • Hainan Prov., Limushan Mt., Limu temple (黎母山黎母庙), 2011.V.5, Shuang Zhao leg.; SYSU.

##### Distribution.

(Fig. 9) China (Hainan), Indonesia, Malaysia, Thailand (Short and Swanson 2005).

### ﻿A key to Chinese species

**Table:** 

1	Elytra with distinct rows of systematic punctures. Pronotum with posterolateral corners angulate (Figs 6 A–C, 7A–C)	**2**
–	Elytra without distinct rows of systematic punctures. Pronotum with posterolateral corners rounded (Figs 6 D–I, 7D–I)	**4**
2	Body length over 5.3 mm (Fig. 7A). Head, pronotum, and elytra with extremely fine and moderately coarse ground punctures. Lateral margins of elytra distinctly expanded (Fig. 6A). Pseudepipleura wide throughout	***O.extensus* sp. nov.**
–	Body length less than 5.2 mm (Fig. 7B, C). Head, pronotum, and elytra with fine ground punctures. Lateral margins of elytra not expanded (Fig. 6B, C). Pseudepipleura posteriorly narrowed	**3**
3	Body length 5.0–5.2 mm. Elytra with 6 irregular rows of large punctures, a short scutellar row of punctures on elytra present (Figs 6B, 7B). Procoxae without spiniform setae	***O.magnificus* Hebauer & Wang**
–	Body length less than 4.5 mm. Elytra with 5 distinct rows of punctures, without scutellar row of punctures (Figs 6C, 7C). Procoxae with distinct spiniform setae	***O.sumatrensis* Orchymont**
4	Abdomen covered only with fine pubescence, without rows of long setae. Aedeagus with gonopore at apex of median lobe (Fig. 8D–F)	**5**
–	Abdomen covered with fine pubescence and rows of long setae. Aedeagus with gonopore below apex of median lobe (Fig. 8G–I)	**7**
5	Meso- and metafemora with fine microsculpture on intervals of punctures. Inner margin of paramere sinuate in dorsal view and distinctly subapically curved; median lobe almost parallel-sided medially, slightly widened at level of gonopore (Fig. 8E)	***O.latiorificialis* sp. nov.**
–	Meso- and metafemora without microsculpture on intervals of punctures. Inner margin of paramere straight or slightly sinuate in dorsal view, not subapically curved; median lobe gradually narrowed from base to apex	**6**
6	Dorsum with distinct greenish luster under lateral illumination. Aedeagus with median lobe shorter than parameres; anterior margin of gonopore rounded (Fig. 8D)	***O.bhutanicus* Satô**
–	Dorsum with weak greenish luster under lateral illumination. Aedeagus with median lobe as long as parameres; anterior margin of gonopore pointed (Fig. 8F)	***O.ximaensis* sp. nov.**
7	Maxillary palps with last palpomere not darkened, punctures on dorsal face finer and sparser. Aedeagus with parameres widely rounded and more or less expanded inwards apically. Median lobe with gonopore situated subapically (Fig. 8I)	***O.dinghu* Short & Jia**
–	Maxillary palps with last palpomere darkened apically, punctures on dorsal face coarser and denser. Aedeagus with paramere narrowly rounded, not expanded inwards apically. Median lobe with gonopore situated at the middle or slightly above the middle	**8**
8	Aedeagus with the outer margin of parameres distinctly curved inwards and clearly narrowed apically; median lobe abruptly narrowed at apical fourth, gonopore wider than long, situated 1.5× its length below apex (Fig. 8H)	***O.shorti* Jia & Mate**
–	Aedeagus with parameres not distinctly curved, and not narrowed apically; medial lobe slightly narrowed subapically, gonopore shaped as a long triangle, longer than wide, situated 1× its length below apex (Fig. 8G)	***O.fikaceki* Short & Jia**

## ﻿Discussion

Three patterns of elytral punctures are known among known species from the Oriental region: 1) elytra with distinct rows of large punctures (including rows of systematic punctures), with ground punctures of almost uniform size, such as *O.sumatrensis* Orchymont, 1932, *O.magnificus* Hebauer & Wang, 1998, and *O.namtok* Short & Swanson, 2005; 2) elytra with a mixture of fine and coarse ground punctures, systematic punctures of almost the same size as coarse ground punctures and interspersed with coarse ground punctures, such as *O.bhutanicus* Satô, 1979, *O.dinghu* Short & Jia, 2011, *O.fikaceki* Short & Jia, 2011, *O.shorti* Jia & Mate, 2012, *O.latiorificialis* sp. nov., and *O.ximaensis* sp. nov.; 3) elytra with distinct rows of large punctures (including rows of systematic punctures), with fine punctures mixed with more coarse ground punctures, such as *O.sitesi* Short & Swanson, 2005, *O.rupicola* Minoshima, 2009 and *O.extensus* sp. nov.

Among Chinese species, two are assigned to pattern 1: *O.sumatrensis*, occurring on Hainan, and *O.magnificus*, endemic to Taiwan. Seven species are assigned to pattern 2: *O.dinghu*, *O.fikaceki*, *O.shorti*, *O.latiorificialis*, *O.bhutanicus*, and *O.ximaensis*, occurring on the Chinese mainland. Only one species, *O.extensus*, is assigned to pattern 3. As in South America, knowledge of the distribution of *Oocyclus* is still very incomplete (Clarkson and Short 2012). In South Asia and Southeast Asia, as well as southern China, many species remain undescribed. A distribution map of the genus in China is presented in Fig. 9. Some provinces in central and western-central China, such as Anhui, Hubei, Chongqing, and Sichuan, receive abundant rainfall and have a suitable climate. Therefore, it is very likely that some unknown species having pattern-2 elytral punctures occur in these regions.

Although *Oocyclus* species are similar in shape, some Neotropical and Oriental groups have evolved some special characters. All 24 Brazilian *Oocyclus* possess pale spots (or “taillights”) on the posterior quarter of the elytra (Clarkson and Short 2012; Jordão et al. 2018; Alencar et al. 2022), apart from the recently described *O.paraiso* from the Guiana Shield, which lacks these spots (Santana et al. 2023). This color feature is found in only two other species of the genus from Venezuela (Clarkson and Short 2012). None of the Oriental species have such a character. Ground punctures in most of the Oriental species vary in size, but only a few Neotropical species have such similar characters. These differences are probably the result of geographical isolation and microenvironment.

The distributions of most species show strong geographic patterns. The pattern of distributions of Venezuelan *Oocyclus* strongly corresponds to mountain ranges and rock outcrops (Short and Garcia 2010). In the fauna of the Neotropical and Oriental regions, only a few species are widely distributed, suggesting that species of *Oocyclus* species may be predominantly local endemics. In China *Oocyclus* species are known to co-occur with species of other genera of Hydrophilidae (*Enochrus*, *Coelostoma*, *Laccobius*), Hydraenidae (*Limnebius*), and Torridincolidae (*Satonius*) (Hájek et al. 2011). Species of these genera, which also inhabit wet rocks or margins of waterfalls, have been collected from Zhejiang Province, Hubei Province, and the Qingling Mountains. The areas in China north of the Yangtze River receive less rainfall and have fewer long-term waterfalls or wet-rock habitats. As a result, no species of *Enochrus*, *Coelostoma*, *Laccobius*, *Limnebius*, or *Satonius*, which inhabit wet rocks or the margins of waterfalls, have been collected there to date.

## Supplementary Material

XML Treatment for Oocyclus extensus

XML Treatment for Oocyclus latiorificialis

XML Treatment for Oocyclus ximaensis

XML Treatment for Oocyclus bhutanicus

XML Treatment for Oocyclus dinghu

XML Treatment for Oocyclus fikaceki

XML Treatment for Oocyclus magnificus

XML Treatment for Oocyclus shorti

XML Treatment for Oocyclus sumatrensis

## ﻿Additional information

### Author ORCIDs

Feng-long Jia https://orcid.org/0000-0003-2391-5038

Zu-qi Mai https://orcid.org/0000-0003-3124-2021

### Data availability

All of the data that support the findings of this study are available in the main text.
